# Giant solitary synovial osteochondromatosis of the elbow causing ulnar nerve neuropathy: a case report and review of literature

**DOI:** 10.1186/1749-7221-8-1

**Published:** 2013-01-25

**Authors:** Munnan Al-Najjim, Abubakar Mustafa, Carl Fenton, Syam Morapudi, Mohammad Waseem

**Affiliations:** 1 Department of Orthopaedics, Macclesfield District General Hospital, Victoria road, Macclesfield, Cheshire, SK10 3BL, UK

## Abstract

**Introduction:**

Giant or solitary osteochondroma is part of a rare disorder known as synovial osteochondromatosis. It forms part of a spectrum of disease characterized by metaplastic changes within the joint synovium that are eventually extruded as loose bodies. It has been suggested that solitary synovial osteochondroma forms as progression of synovial osteochondromatosis through a process of either coalescence of multiple smaller bodies or the growth of a dominant synovial osteochondroma. Previous studies have shown that it occurs as a late phase of the disease. We report a rare case of giant synovial osteochondromatosis at the elbow causing ulnar nerve neuropathy and mechanical symptoms which has not been previously reported in the literature.

**Case report:**

We report a case of a 56 year old Western European gentleman who presented with ulnar nerve neuropathy and swelling behind the elbow. The patient underwent MR imaging and subsequent biopsy that demonstrated synovial osteochondromatosis. Initially the patient declined surgery and opted for a watch and wait approach. Five years later he returned with worsening symptoms and underwent successful surgical resection of a giant solitary synovial osteochondroma.

**Conclusion:**

The unique outcome in our patient despite the long interval between presentation and surgical treatment resulted in early full resolution of symptoms within a short period. It may suggest an improved prognosis as compared to multiple synovial osteochondromatosis in terms of mechanical and neurological outcomes.

## Introduction

Synovial osteochondromatosis is a rare condition characterized by cartilaginous metaplastic changes within the synovium of the joint that eventually ossify and are extruded into the joint space as loose bodies. It is more common in men and usually occurs in the third to fifth decades of life [1]. The knee joint is most commonly affected followed by the hip, elbow, shoulder and ankle [2]. The aetiology of osteochondromatosis remains unproven, however, Mueller et al [3] found that it usually affects the dominant extremities, leading to the hypothesis that biomechanical stress play a role in its’ development. However, the same study suggested that there might be hereditary transmission of the condition.

According to Milgram [4] synovial osteochondromatosis has three phases. Phase 1- active intrasynovial disease with no loose bodies; phase 2- the transitional stage involving active intrasynovial disease and loose body; and phase 3- multiple osteochondral loose bodies without active synovial disease. An additional fourth phase was suggested by Edeiken et al [2] that comprises of a large intra or extra-articular calcified cartilogenous mass, which can be formed by the fusion of multiple synovial chondromas or by the growth of a solitary synovial chondroma.

Synovial chondromatosis usually presents with arthritic symptoms, chronic pain and swelling, clicking and limitation of movement in the affected joint. Infrequently it presents with neurological symptoms; peripheral nerve compression in the elbow caused by synovial chondromatosis is particularly rare.

Diagnosis is based on preoperative radiology including plain radiographs and MRI in addition to intraoperative and histological findings. In early stages radiography may be equivocal especially on plain film where there may be no visible abnormality [5].

Ulnar nerve neuropathy associated with multiple synovial osteochondromatosis of the elbow has been reported [6-10].

We report a rare case of giant synovial osteochondromatosis at the elbow causing ulnar nerve neuropathy and mechanical symptoms, which has not been previously reported in the literature.

## Case report

A 56 year old, right hand dominant Western European engineer was referred in 2004 with a history of swelling behind his right elbow for 2 years. The size of the swelling tended to fluctuate and more recently he had begun to develop intermittent pain and paraesthesia in the ulnar nerve distribution. There was no associated history of trauma and function of the elbow was largely unaffected.

Clinical examination revealed a 3 × 2 cm swelling on the posteromedial aspect of the elbow. It was firm with a well defined margin and smooth surface. The ulnar nerve could be palpated overlying the mass. Mobility of the mass was reduced on contraction of the triceps which suggested deep tethering. The overlying skin was free. There was no muscle wasting or clinically detectable distal neurovascular deficit.

**Figure 1:**
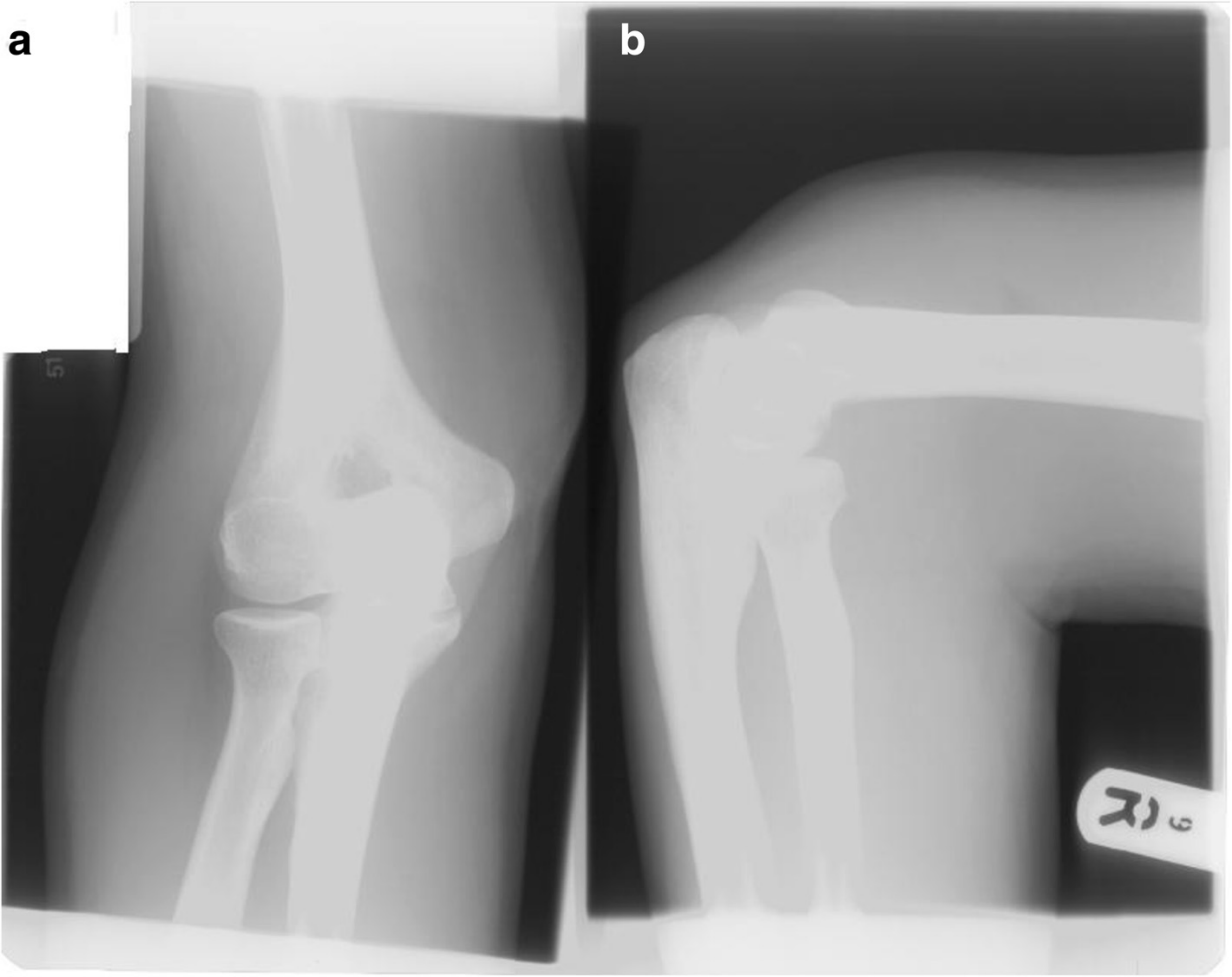
(a) Lateral and (b) anteroposterior radiographs of the right elbow demonstrating no visible abnormality.

Initial plain radiographs of the elbow demonstrated no abnormalities (Figure 1). The patient was evaluated with urgent Magnetic Resonance Imaging (MRI) that showed a well circumscribed mass at the posterior capsule of the joint with high signal on T2 weighted images and notably, no evidence of bony destruction which suggested a benign pathology, possibly synovial osteochondromatosis. A biopsy was performed which confirmed osteochondromatosis with active intra-synovial disease.

The swelling remained unchanged over the next 6 months, so he declined surgery and opted for a watch and wait approach. Although the risks of the disease including malignant transformation were discussed with the patient, he was reluctant to undergo surgical intervention and requested discharge from the clinic.

**Figure 2:**
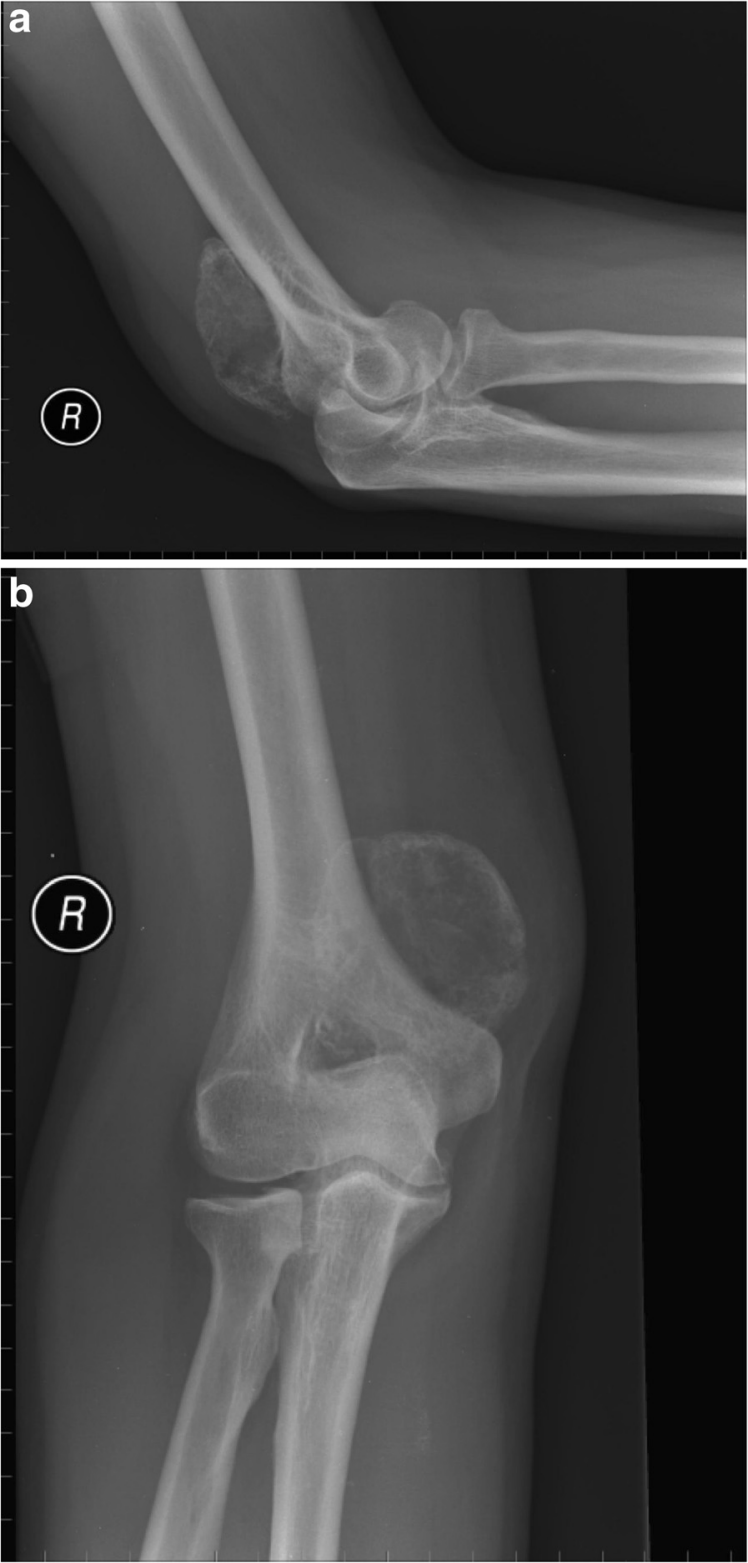
(a) Lateral and (b) anteroposterior radiographs of the right elbow demonstrating a solitary well defined calcified mass at the posteriomedial aspect.

**Figure 3:**
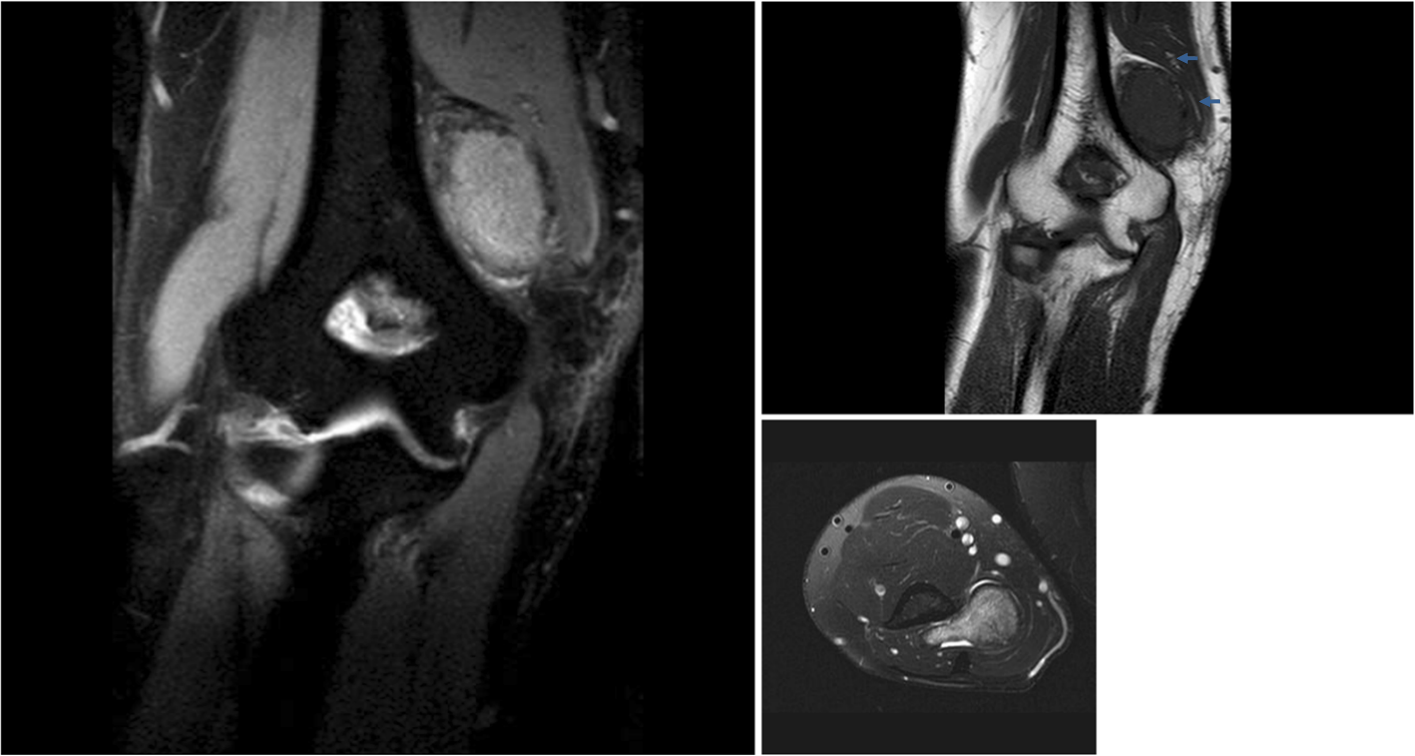
(a&b) MR coronal and (c) axial images showed well defined mass above the medial epicondyle with ulnar nerve compression (arrowed).

Five years later he returned complaining of increasing paraesthesia in the ulnar nerve distribution, additionally, there was worsening pain referring back to the brachial plexus and limited range of motion. Clinical examination revealed a 5 × 5 cm mobile mass with no skin tethering. His range of motion was 15 to 100 degrees, supination and pronation were normal. Tinel’s sign was positive. Plain radiographs and MR scan (Figures 2 and 3) corroborated the diagnosis of synovial chondromatosis; it also showed that the ulnar nerve was markedly displaced and compressed by the lesion at the level of olecranon fossa, however the cubital tunnel was normal. Nerve conduction studies did not demonstrate significant deficit.

**Figure 4:**
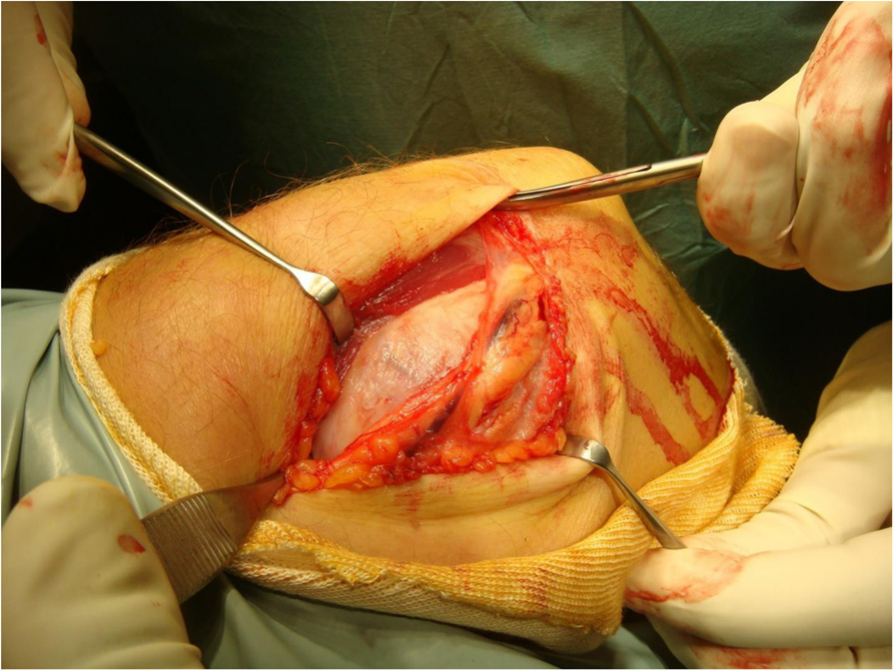
The lesion in situ at operation with the ulnar nerve visible was overlying the mass.

**Figure 5:**
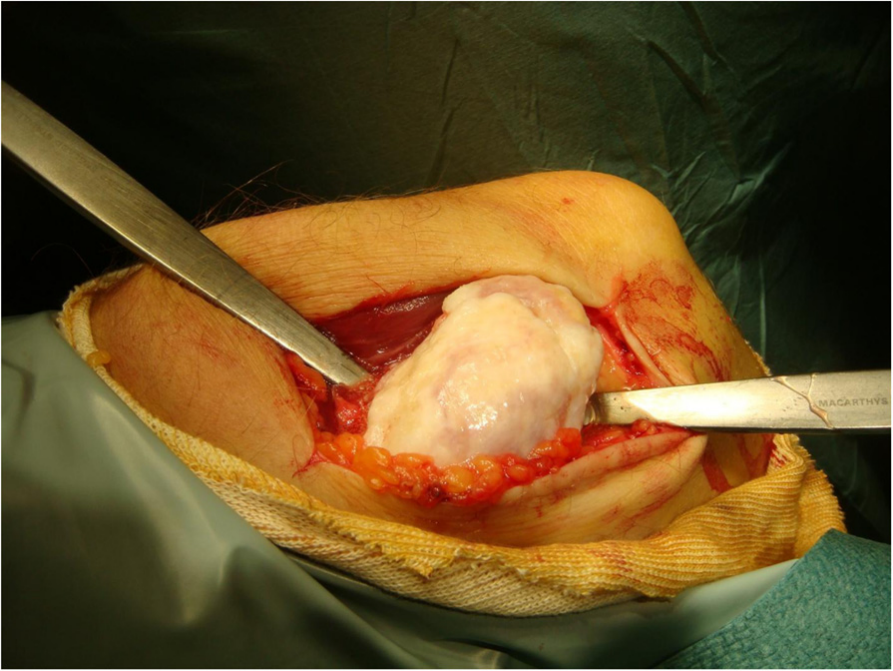
Shows the mass in situ.

**Figure 6:**
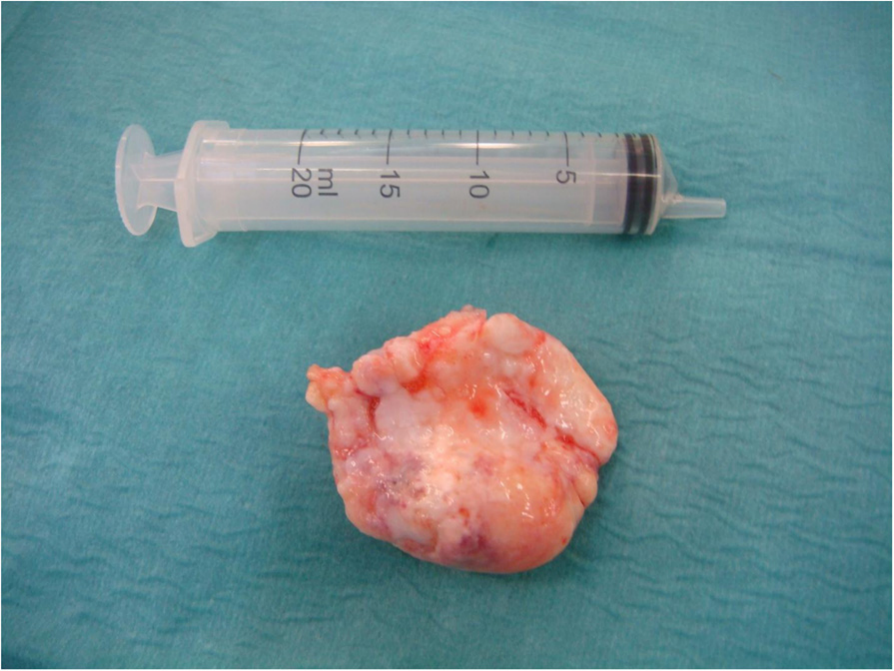
The mass after excision.

This time the patient opted for surgical intervention. Through a posteromedial approach centered over the mass, the ulnar nerve was identified and protected (Figure 4). The joint capsule was opened and the mass removed (Figures 5 and 6). There was no obvious active intra-synovial disease and it was classified as phase 3, therefore synovectomy was not performed. The ulnar nerve was released proximally and distally. No transposition of the nerve was performed as the previous radiographic studies has demonstrated a normal cubital tunnel and the nerve was free following removal of the mass and decompression. There was no soft tissue invasion or bony destruction. Post-operative recovery was uneventful. Histopathological examination confirmed osteochondromatosis with no active intra-synovial disease.

Six weeks post operatively, the patient had resumed normal activity and returned to work. There was complete resolution of both neurological and mechanical symptoms at three months post operatively. At two years follow-up the patient remains symptom free with full range of movement at the elbow.

## Discussion

Although osteochondromatosis is considered to be a benign condition, some studies suggest that around 5 percent of cases may undergo transformation to chondrosarcoma [11,12]. Clinically and radiologically it is very difficult to distinguish a giant solitary synovial chondroma from chondrosarcoma, parosteal osteosarcoma or osteochondroma [2,13]. Edeiken et al [2] documents the radiological appearance of giant synovial osteochondromatosis in ten cases affecting 5 different joints including one case affecting the elbow. The classical plain radiographic appearance of the multiple synovial osteochondromatosis is multiple oval, well defined, intra-articular calcified loose bodies and varying from few millimeters to 1 cm in diameter. Calcification is present in up to 66% of the cases and can be diagnosed by plain radiograph. In radiolucent lesions, ultrasonography [14], arthrography, CT, arthro-CT [15] or MRI scanning as in our case has a role in detecting lesion not diagnosed by the plain radiograph.

Coalescent of multiple synovial osteochondromatosis may give rise to giant chondroma causing a feathery radio-opaque appearance on plain radiograph; erosion of the adjacent bone may be present which might mimic a malignant condition, therefore, histological examination is essential to distinguish between benign and malignant growth. However, one synovial osteochondroma may grow independently to reach a size more than 1 cm. occasionally a mixture of coalescent and multiple osteochondromatosis can occur.The single elbow case documented by Edieken et al was a result of a coalescent of multiple osteochondromatosis and they did not elucidate on any mechanical or neurological sequelae of the disorder.

The presence of a giant synovial osteochondroma has been classified as a fourth stage of the disease [2], however, as our patient demonstrates giant synovial osteochondroma may be present in both stages 2 and 3 indicating that this is not a progression but a subset of those suggested by Milgram [4].

Treatment of synovial chondromatosis depends on the stage of disease at presentation. The mainstay of treatment is surgery to alleviate symptoms and consists of removal of loose bodies along with excision of any active, involved synovium [6], which may prevent recurrence [3].

Giant solitary synovial osteochondroma of the elbow with ulnar nerve neuropathy has not been reported in the literature. There are few reported cases of multiple synovial osteochondromatosis with ulnar nerve neuropathy [6-10]. Kim et al noted incomplete resolution of neurological symptoms 15 months following the excision of 3 years old mass. Ruth and Groves reported full resolution of neurological symptoms but reduced range of movement 30-110 degrees following the removal of 2 years old mass. Fahmy and Noble documented a similar case of 5 years history with ulnar nerve palsy and mechanical restriction with a range of motion 40–130 degrees. Neurological symptoms improved over a 6 months period and the excision allowed the full range of movement to be regained. Nogueira et al described a case of 1 year history with ulnar and median nerve neuropathy. No mechanical symptoms were noted. After surgery, the time for full neurological recovery was 3 months for the median nerve and 3 years for the ulnar nerve. Kamineni et al presented 12 cases with only 3 of these having both mechanical and ulnar nerve symptoms, improvement of the mechanical symptoms was achieved over a period of time between 6 and 9 years but this was incomplete. Recovery from the neurological symptoms was reported in only one case.

It is apparent from the published data that multiple synovial osteochondromatosis may show delayed and incomplete recovery of symptoms following surgery for either the mechanical or the neurological symptoms or both.

To our knowledge, there is no documented report of a case of giant solitary synovial osteochondroma with ulnar nerve neuropathy with full recovery and early return to normal daily activity as in our patient.

## Conclusion

This case highlights an interesting presentation of a rare disorder with the formation of a giant synovial osteochondroma within the earlier stages of the disease not reported previously in the literature. In our patient surgical intervention, even after a long delay, resulted in early full resolution of symptoms within a short period. It may suggest an improved prognosis as compared to multiple synovial osteochondromatosis in terms of mechanical and neurological outcomes despite delayed presentation.

## Consent

Written informed consent was obtained from the patient for publication of this case report and any accompanying images. A copy of the written consent is available for review by the Editor-in-Chief of the journal.

## Competing interests

The authors declare that they have no competing interests.

## Authors’ contributions

MA drafted the manuscript, reviewed the literature and made major contributions to the writing of the manuscript. AM and CF helped draft the manuscript, reviewed the patient in the clinic and contributed to the writing of the manuscript. SM took the photographs and also reviewed the patient in the clinic. MW reviewed the patient in the clinic, carried out the operation, read and approved the final manuscript. All authors read and approved the final manuscript.
